# Inhibition of lipopolysaccharide-induced inflammation by trophoblast-conditioned medium and trophoblast-derived extracellular vesicles in human middle ear epithelial cells

**DOI:** 10.1038/s41598-023-46731-7

**Published:** 2023-11-14

**Authors:** Chan Mi Lee, Yoon Young Go, Jae-Jun Song

**Affiliations:** 1 Division of Otorhinolaryngology-Head and Neck Surgery, Korea University College of Medicine, Seoul, Republic of Korea; 2 Center for Health Care Convergence at Korea University Guro Hospital, Seoul, Republic of Korea

**Keywords:** Molecular biology, Stem cells

## Abstract

Otitis media is a common disease but can cause severe inner ear inflammation and hearing loss if it persists for more than two weeks. This study elucidates the inflammation-inhibiting efficacy of conditioned medium (CM) and extracellular vesicles (EVs) derived from human trophoblast (TB) cells in lipopolysaccharide (LPS)-induced human middle ear epithelial cells (HMEECs). TB-conditioned medium (TB-CM) reduced the inflammatory response and regulated mucin and epithelial sodium channel genes in LPS-induced HMEECs. The underlying mechanism of cell migration during inflammatory healing in LPS-induced HMEECs treated with TB-CM was determined by RNA-sequencing analysis. Specifically, the NF-κB pathway related to the copper metabolism MURR1 domain protein was studied and verified through siRNA. This elucidation of the anti-inflammatory effect of TB-CM and TB-derived EVs demonstrates their clinical potential to treat chronic inflammation.

## Introduction

Otitis media is a common condition that 80–90% of children develop once or twice before the age of 7 before the internal organs of the ear are fully developed^1^. Acute otitis media manifests within a week, but if it persists for over 2 weeks, it progresses into chronic otitis media^2^. Chronic otitis media can cause severe inner ear inflammation and hearing loss. There are drug and surgical treatments for otitis media; however, drugs are not effective for the long-term treatment of chronic otitis media^3,4^. Novel remedies to manage and cure otitis media are widely studied in the pathobiology and anti-inflammatory substance fields. Human stem cells from diverse sources, such as human tissue derived from MSCs, bone marrow, and placenta, have been identified as effective remedial agents^5,6^.

Mesenchymal stem cells (MSCs) present potential advantages as treatment; however, their use for immediate cell treatment can result in adverse reactions, such as swelling and the growth of abnormal tissue in the receiver. Therefore, Cell-free biomaterials that have anti-inflammatory efficacy include cell-derived conditioned medium (CM) and extracellular vesicles (EVs), such as exosomes. EVs are composed of a 100–1000 nm phospholipid bilayer and are classified into three main types: microvesicles, ectosomes, or exosomes^6^. Exosomes functionally mediate cell–cell interactions and cellular immunity and deliver membrane components, proteins, and RNA to other cells and tissues^8^. Exosomes transported from stem cells contain various stem cell-secreted growth factors and cytokines and regulate cell adhesion, growth, and differentiation. Human umbilical cord MSC-derived exosomes assist skin wound healing with increased expression of PCNA, CK19, and type I collagen^9,10^. MSC-derived exosomes modulate the action of macrophages, inhibit NF-ĸB P65 nuclear translocation, and reduce neurotoxic A1 astrocyte activation, enforcing neuroprotection^11^. Furthermore, MSC-derived EVs improve motor activity via inflammation mitigation in the umbilical cord tissue and is characterized by disordered astrocytes and microglia^12^.

Among various stem cell sources, placental stem cells have low immunogenicity characteristics, the capability to transform into all three germ layers, and the expression of pluripotency markers^13^. The placenta has the advantage that it is generally disposed of and thus, has no supply or ethical-related issues^14^. Placental chorion-derived MSCs have notable proliferation and immune regulation properties^15^. For the study of the placenta, trophoblastic, chorionic, mesenchymal, and amniotic epithelial cells can be isolated as cell types^14,16^. Trophoblasts (TBs) transmit maternal signals to the fetus by secreting various growth factors, biological signaling substances, and hormones (including EVs, such as microvesicles and exosomes)^17^. TBs deliver nutrients and remove waste products that are necessary for embryo maturation and development during pregnancy. This transfer occurs in both directions (between the mother and the fetus) and facilitates metabolism related to the synthesis or secretion of various substances within the placenta^18^.

However, no prior investigations have evaluated the anti-inflammatory impacts of TB-derived culture media and EVs on human middle ear epithelial cells (HMEECs) yet. Lipopolysaccharide (LPS) is a constituent of the cell envelope of gram negative bacteria^19^. Toll-like receptor 4 (TLR4) signaling via the myeloid differentiation primary response gene 88 (MYD88) pathway is an important pathway to combat bacterial infection and also causes otitis media^20^. The TLR4/MyD88 pathway is essential in repairing inflammation of the middle ear^19,20^. Therefore, in this study, we assessed the anti-inflammatory effects of TB-derived EVs and CM in LPS-induced HMEECs. We propose RNA-sequence profiling to demonstrate the changes induced by the TB-CM and elucidate the novel working mechanism.

## Results

### In vitro inflammation model in HMEECs by LPS

The accessible nature of HMEECs allowed us to evaluate anti-inflammatory effects in otitis media in vitro. We observed the morphology (Fig. S1a) and cell viability (Fig. S1b) of HMEECs after treatment with LPS in a concentration-dependent manner (0, 2.5, 3.5, and 5.6 μg/mL) for 24 h. In cell viability, half maximal inhibitory concentration (IC_50_) was determined to be 5.67 ± 0.25 μg/mL, and cell viability decreased in an LPS concentration-dependent manner (Fig. S1c). To measure inflammatory factors, the *tumor necrosis factor-α (TNF-α)* and *cyclooxygenase-2 (COX-2)* mRNA expression levels were measured by quantitative real-time polymerase chain reaction (RT-qPCR). The results indicated that the mRNA expression levels increased in an LPS concentration-dependent manner (Fig. S1d).

### TB-CM suppresses inflammatory cytokines in LPS-induced HMEECs

**Figure 1:**
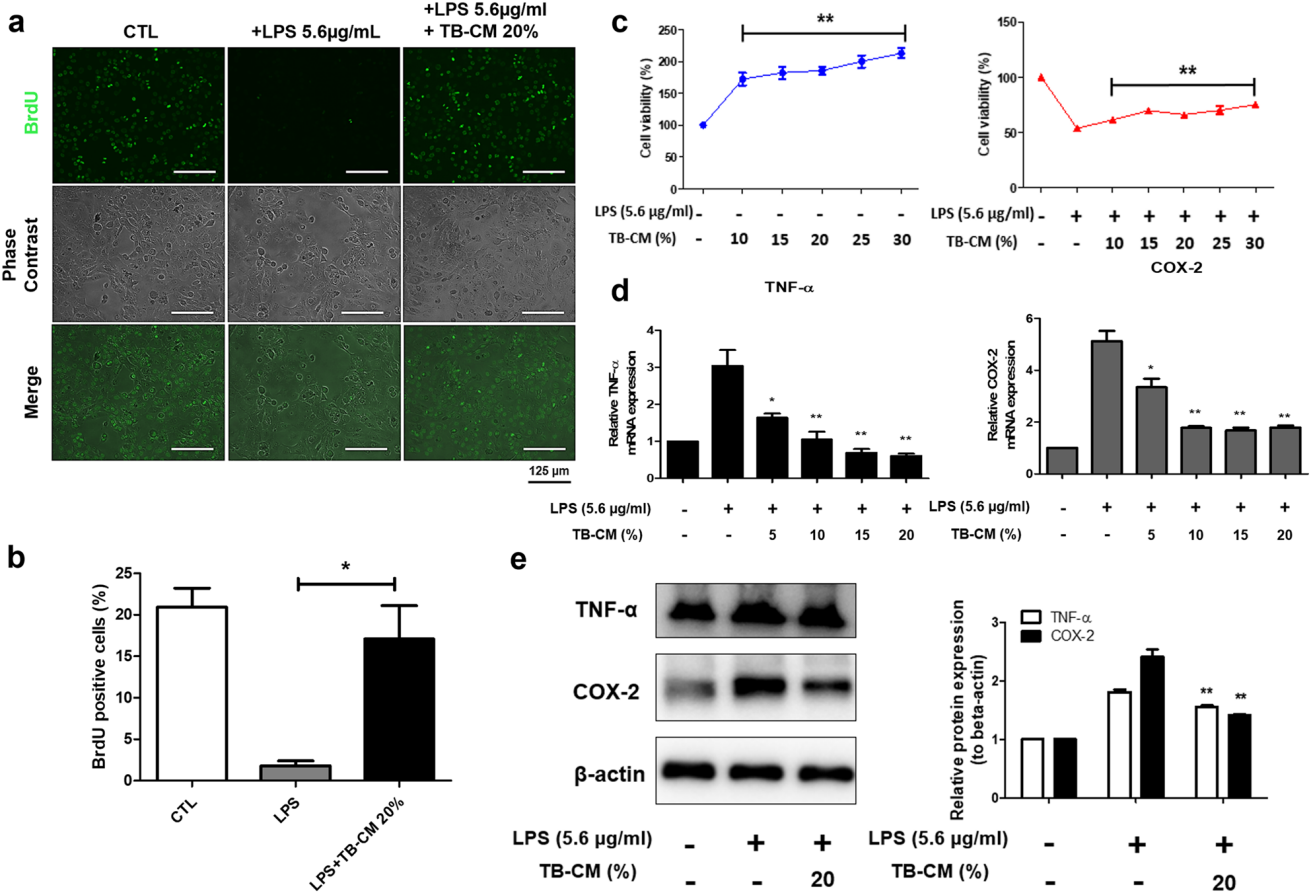
Trophoblast-conditioned medium (TB-CM) increased cell viability and decreased inflammatory marker mRNA expression levels (**a**) Immunofluorescence analysis of BrdU incorporation (Green) was performed by culturing HMEECs after treatment with TB-CM for 24 h in the presence or absence of LPS. The scale 125 µm. (**b**) The BrdU positivity rate was examined for comparison. (**c**) Cell viability was measured using CCK-8, and in HMEECs, the LPS-treated group showed approximately 50% cell death compared to the control group. Cell viability increased in a TB-CM concentration-dependent manner. (**d**) *TNF-α* and *COX-2* mRNA expression levels decreased in a concentration-dependent manner to TB-CM. (**e**) Western blot analysis of inflammatory markers (TNF-α and COX-2). Western blots were quantified using Image J software as a densitometric method. All statistics are provided as the mean of three independent experiments. *P < 0.05, **P < 0.01, and ***P < 0.001.

TB-derived cells possess various elements associated with cellular growth and compounds that mitigate inflammation^18,21,22^. Therefore, we hypothesized that TB-CM contains anti-inflammatory factors and compared the efficacy of TB-CM treatment after inducing inflammation with LPS in HMEECs. Cell proliferation was reduced in the LPS group compared to the control group in the BrdU-positive reaction. However, in the TB-CM treatment group, the BrdU incorporation was similar to that in the control group (Fig. 1a). BrdU-positive cells (%) were quantified in the control group (20.941 ± 3.225), LPS group (1.805 ± 0.806), and TB-CM group (17.085 ± 5.702). TB-CM group exhibited a significant increase in BrdU-positive cells when compared to the LPS group (Fig. 1b). Cell viability increased in TB-CM treated cells in a concentration-dependent manner, in the presence or absence of LPS (Fig. 1c). Thus, we observe that TB-CM affects HMEEC proliferation and viability. To estimate the mRNA and protein expression of the inflammatory cytokine markers, TNF-α and COX-2, we used RT-qPCR or Western blot, respectively. mRNA expression levels confirmed the decrease of inflammatory markers in a concentration-dependent manner with TB-CM treatment (5, 10, 15, or 20%) (Fig. 1d). Western blot analysis confirmed that TNF-α and COX-2 protein expression levels were significantly reduced in 20% TB-CM treated group compared to the LPS group (Fig. 1e). Furthermore, to identify the optimal TB-CM concentration, we evaluated COX-2 protein expression using Western blot analysis with TB-CM (20% and 30%). It was observed that while 20% TB-CM resulted in a decrease compared to LPS, 30% TB-CM led to an increase. As a result, we concluded that 20% TB-CM was the optimal concentration and consistently utilized it in our experiments (see Fig. 1e in Supplementary Fig. S4). These findings predict that 20% TB-CM reduces the inflammatory response in LPS-stimulated HMEEC.

### TB-CM regulates water channels in LPS-induced HMEECs

**Figure 2:**
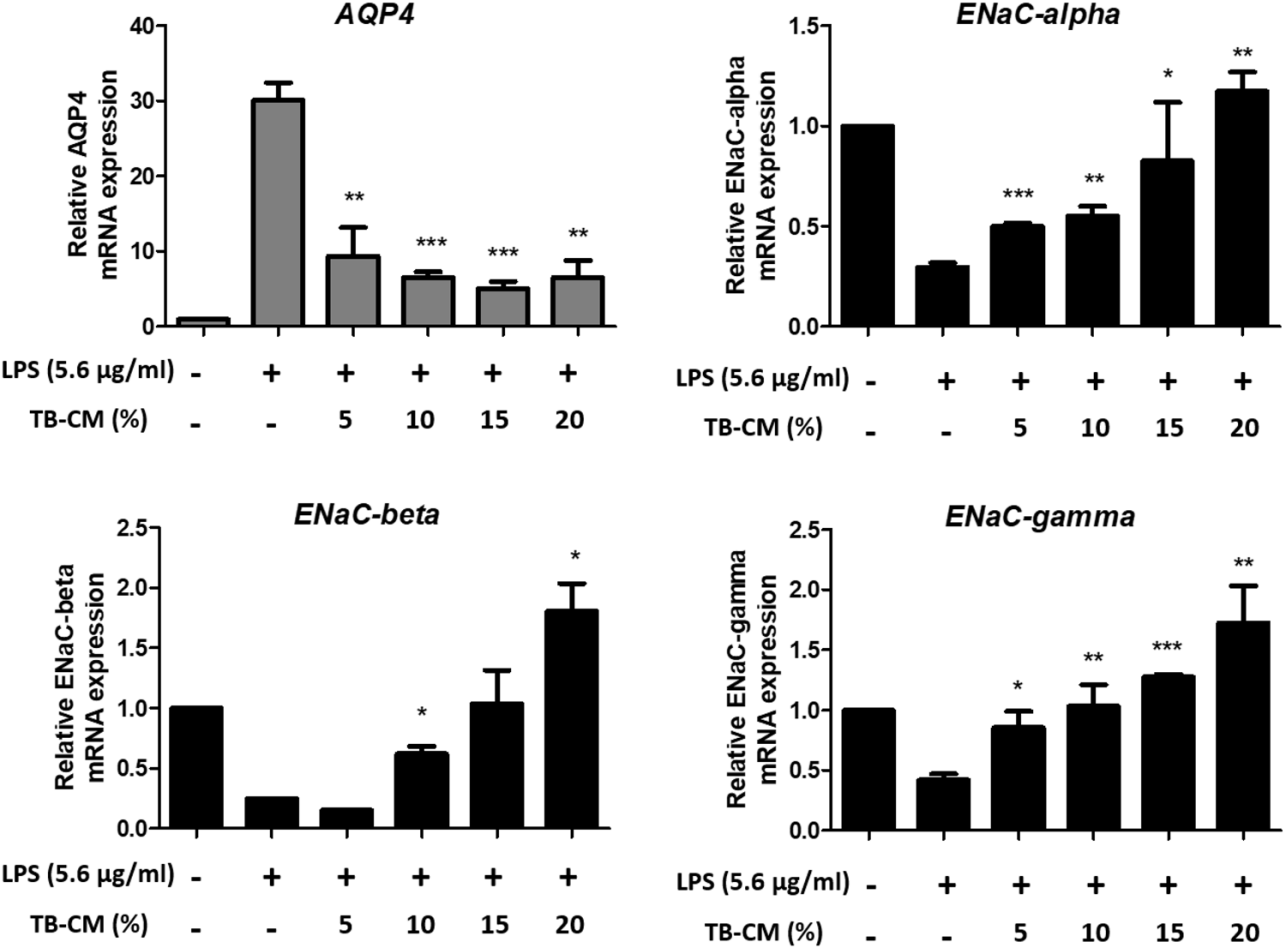
Trophoblast-conditioned medium (TB-CM) reduced water channel dysregulation caused by lipopolysaccharide (LPS) in human middle ear epithelial cells (HMEECs). TB-CM was treated in a concentration-dependent manner to obtain quantitative values for *AQP4* and *ENaC* family genes using real-time polymerase chain reaction for the expression levels of aqueduct-related genes. TB-CM decreased the *AQP4* gene and increased the *ENaC* family compared to the LPS group. The data is displayed as mean ± standard deviation (n = 3). In comparison to the control group, the significance levels were *P < 0.05, **P < 0.01, and ***P < 0.001.

HMEECs possess water channels or aquaporin (AQP) and epithelial Na^+^ channel (ENaC)^23^. Since AQP4 is involved in the transport of water by osmotic pressure in the middle ear, gene expression levels were evaluated upon LPS-induced inflammation^23,24^. The mRNA expression levels of *AQP4* in LPS-stimulated HMEECs increased compared to the control group and decreased in TB-CM treated cells in a concentration-dependent manner. Na^+^, the most concentrated electrolyte, moves through ENaC into the extracellular fluid and is a major determinant of the osmotic pressure of the extracellular fluid^25,26^. Thus, the middle ear cavity retains dehydrated air so sound can be transmitted effectively^27^. In HMEECs, upon LPS-induced inflammation, mRNA expression levels of the *ENaC* family genes (α, β, and γ) were found to decrease in the LPS group and increase in TB-CM treated cells in a concentration-dependent manner (Fig. 2).

### TB-CM inhibits mucin production in LPS-induced HMEECs

**Figure 3:**
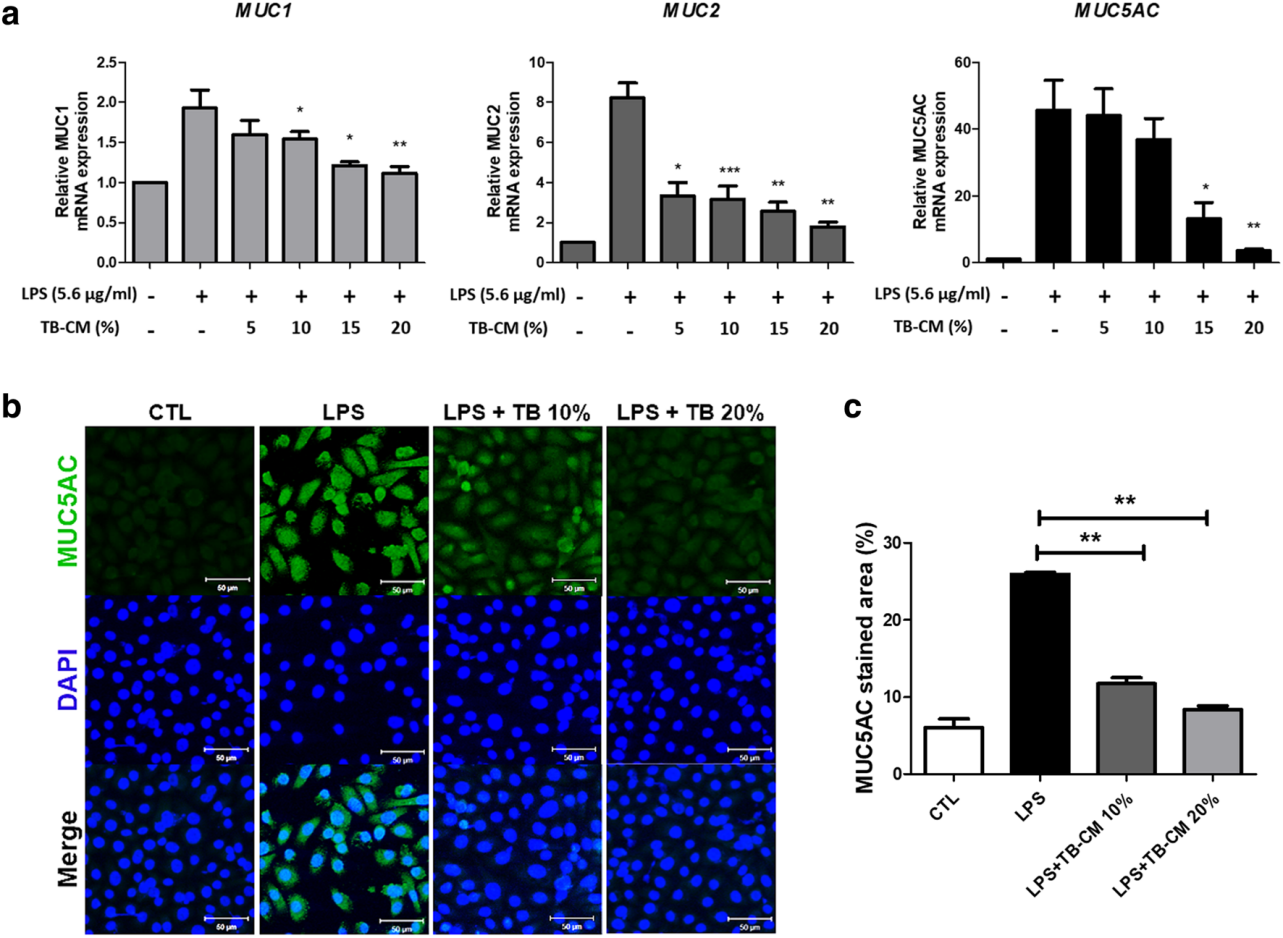
Trophoblast-conditioned medium (TB-CM) reduced mucus production in lipopolysaccharide (LPS)-induced human middle ear epithelial cells (HMEECs). (**a**) HMEECs were treated to half inhibitory concentration (IC_50_) for 24 h and the quantitative real-time polymerase chain reaction technique was used to examine the mucin gene expression levels. The mRNA expression levels of *Muc1*, *Muc2*, and *MUC5AC* genes were significantly reduced. (**b**) Immunofluorescence staining for MUC5AC expression (green) and nuclei (blue) counterstained with 4′,6-diamidino-2-phenylindole (DAPI). MUC5AC levels were lower in the TB-CM group. Scale bar 50 µm. (**c**) MUC5AC stained area (%) was quantified using image J (*P < 0.05, **P < 0.01, and ***P < 0.001).

Excessive mucus secretion or characteristic changes can exacerbate inflammation, so it is crucial to control the excessive secretion of mucus^28–30^. The inflammatory response in HMEECs was measured by RT-qPCR, by analyzing mRNA expression levels of genes that encode mucins. In the present study, we showed that in LPS-treated HMEECs, the LPS group showed approximately 1.92-, 8.22-, and 45.62-fold increases in the mRNA levels of *MUC1*, *MUC2*, and *MUC5AC*, respectively, compared to the control group (Fig. 3a). Treatment with TB-CM markedly reduced mRNA levels in a concentration-dependent manner. Immunofluorescent staining of MUC5AC expression in HMEECs showed that MUC5AC levels are significantly increased in LPS-induced cells, and treatment with TB-CM decreased MUC5AC levels. These results indicated that TB-CM treatment reduces mucin production in inflamed HMEECs (Fig. 3b). MUC5AC stained area (%) was quantified using ImageJ and the results were as follows: Control group (6.002 ± 1.651), LPS group (25.937 ± 0.314), 10% TB-CM group (11.741 ± 1.093), and 20% TB-CM group (8.362 ± 0.714). Importantly, MUC5AC exhibited a significant dose-dependent decrease in comparison to the LPS group as the TB-CM concentration increased (Fig. 3c).

### Transcriptome changes of TB-CM using RNA-sequencing

**Figure 4:**
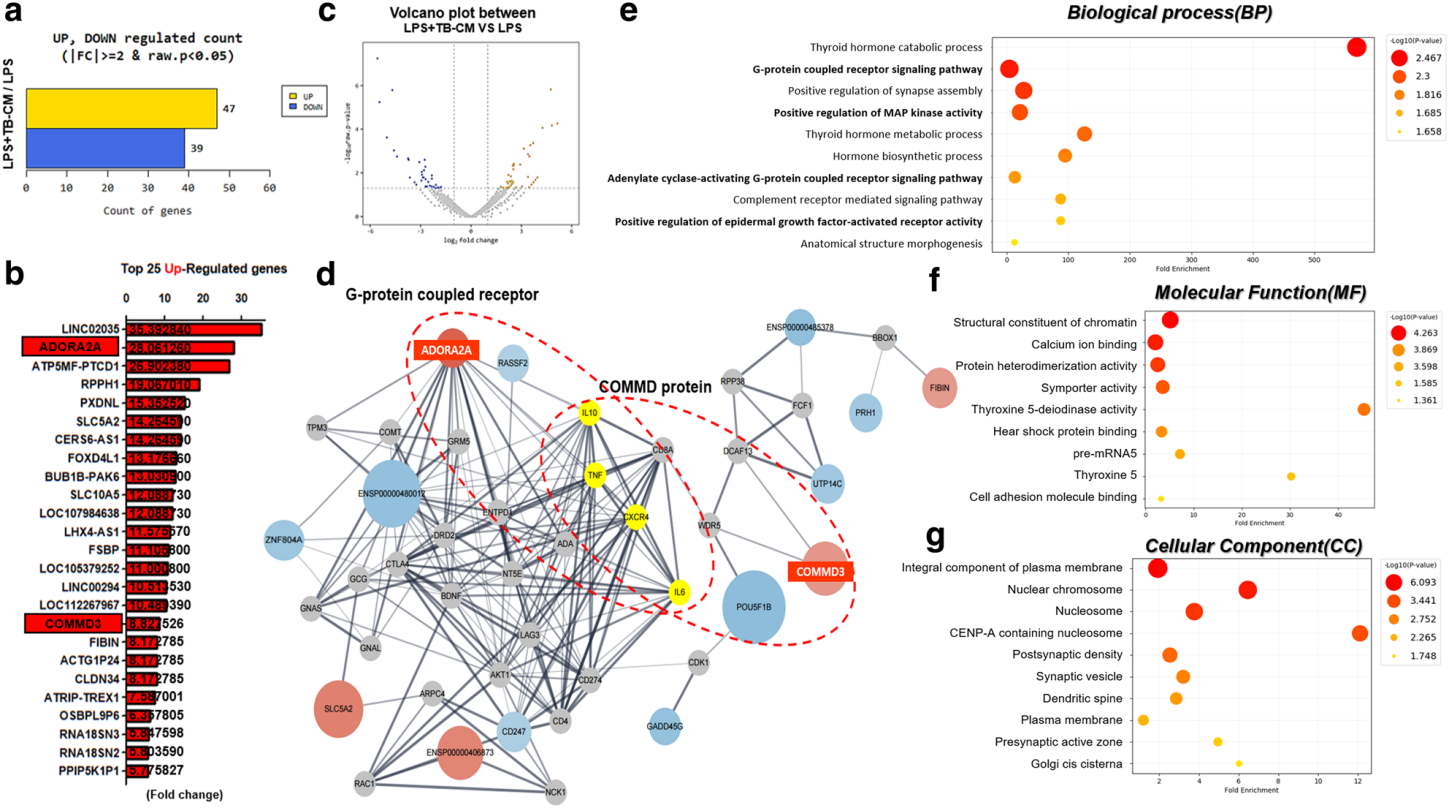
Trophoblast-conditioned medium (TB-CM) induced transcriptome changes in human middle ear epithelial cells (HMEECs). (**a**) Differentially expressed genes (DEGs) were detected by performing RNA-seq analysis on HMEECs, comparing the lipopolysaccharide (LPS) group with the LPS + TB-CM group. The results revealed that 47 genes were upregulated, and 39 genes were downregulated. (**b**) The top 25 upregulated genes in TB-CM-treated HMEECs are color images and fold change values. (**c**) Volcano plots were visualized in the form of significantly increased and decreased plots with different colors, and the fold change value was set to 2 and displayed. (**d**) The Cytoscape tool was used to visualize the prediction results of the protein–protein interaction (PPI) network between COMMD3 and ADORA2A among upregulated genes. (**e**–**g**) Gene ontology (GO) enrichment analysis of DEGs. The X-axis is the fold enrichment value, and the higher the P-value, the darker the red color is displayed. All analyses were performed at |FC|≥ 2 and P < 0.05.

DEseq2 was used to screen differentially expressed genes (DEGs) among the LPS and LPS + TB-CM groups. A total of 86 differential genes (FC ≥ 2, P < 0.05) were obtained, of which 39 were downregulated and 47 were upregulated (Fig. 4a). Upregulated genes are shown through a graph by selecting the top 25 genes. *ADORA2A* was upregulated 28.06-fold and *COMMD3* 8.82-fold (Fig. 4b). DEGs between groups were visualized on a volcano plot. Upregulated genes are shown in yellow and downregulated genes in blue (Fig. 4c). We visualized the protein–protein interaction (PPI) network of upstream or downstream genes. Each node represents a protein, and each edge thickness reflects the strength of the PPI network. Red nodes indicate genes with accelerated expression, and blue suggests genes with decreased expression. The yellow node is a gene related to inflammation, and the interaction of *IL-10*, *IL-6*, *TNF*, and *CXCR4* genes related to COMMD protein and G protein-coupled receptor ADORA2A was confirmed (Fig. 4d). Gene ontology (GO) analysis was done using DAVID from three perspectives: biological process (BP) (Fig. 4e), molecular function (Fig. 4f), and cellular component (Fig. 4g). The COMMD protein was especially considered among the upregulated genes by RNA-seq analysis.

### TB-CM regulates COMMD and NF-ĸB in LPS-induced HMEECs

**Figure 5:**
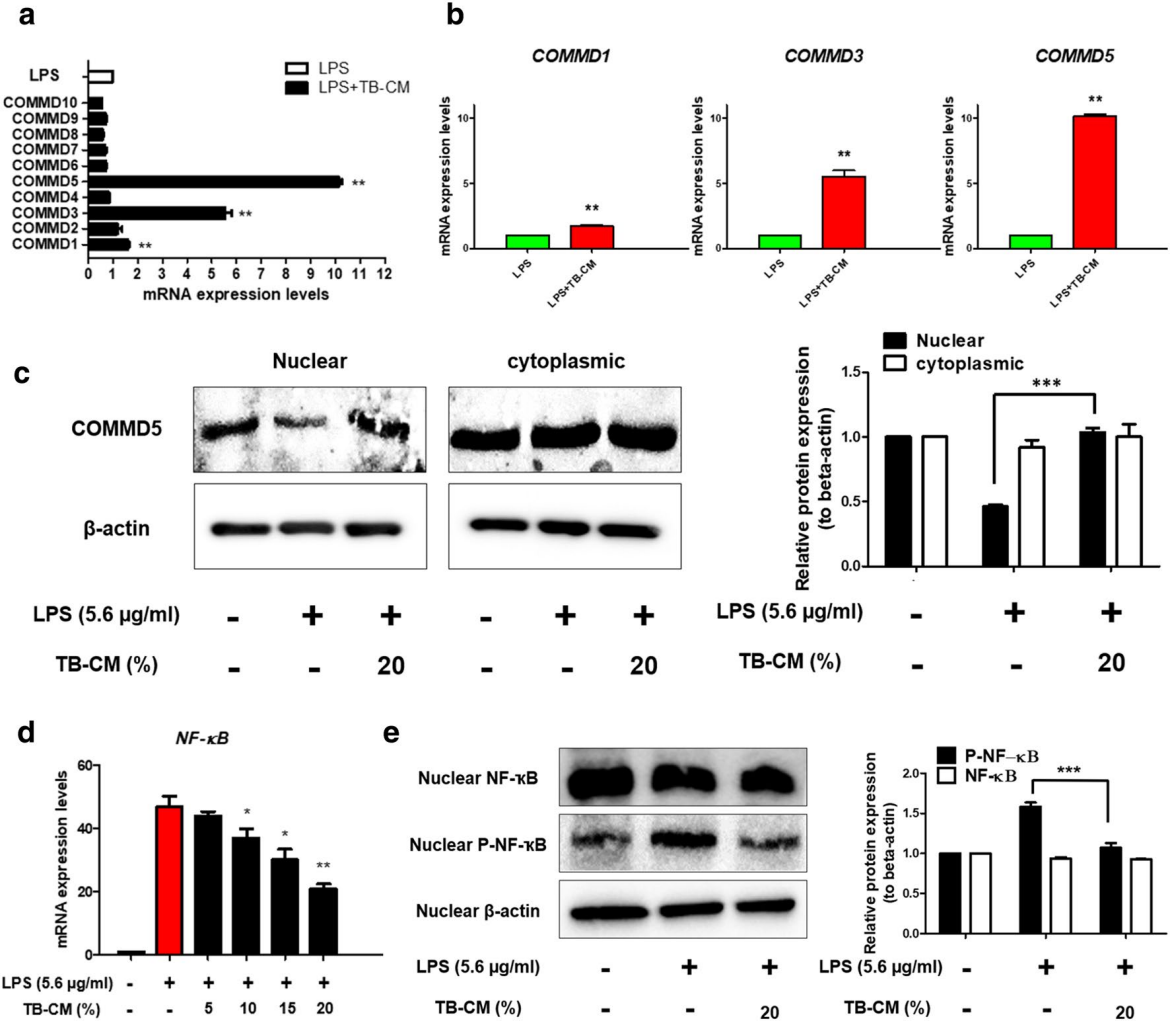
Regulation of copper metabolism MURR1 domain (COMMD) and nuclear factor (NF)-ҡB by trophoblast-conditioned medium (TB-CM) in lipopolysaccharide (LPS)-induced human middle ear epithelial cells (HMEECs). (**a**) mRNA expression levels of the *COMMD* family (1–10) genes were examined by quantitative real-time polymerase chain reaction. (**b**) Significant upregulation was confirmed in the mRNA expression levels of *COMMD1*, *COMMD3*, and *COMMD5*. (**c**) Western blot analysis using an antibody of COMMD5 was used, which indicated the highest mRNA expression levels in the HMEEC nucleus and cytoplasm. There were no notable differences in the levels of COMMD5 protein expression in the cytoplasm. However, the protein expression levels of cell nuclei decreased and increased in the LPS and TB-CM groups, respectively. (**d**) mRNA expression levels of *NF-ҡB* decreased in a TB-CM concentration-dependent manner in LPS-induced HMEECs. (**e**) Proteins from the nucleus of cells were separated and subjected to Western blot analysis using NF-ҡB, pNF-ҡB, and β-actin antibodies. While the protein expression levels of pNF-ҡB increased in LPS-induced HMEECs, the protein expression level of pNF-ҡB significantly decreased when treated with TB-CM. β-actin expression levels were used to normalize all Western blot data. The outcomes of three separate trials are displayed as average ± standard deviation (SD). *P < 0.05, **P < 0.01, and ***P < 0.001.

We constructed primers for COMMD1-10 (Table S1) and analyzed mRNA expression by RT-qPCR (Fig. 5a). Compared to the LPS group, the LPS + TB-CM group showed significantly upregulated mRNA expression levels of *COMMD1*, *COMMD3*, and *COMMD5* (Fig. 5b). Cytoplasm and nucleus were isolated from HMEECs of each group, and protein expression was expressed by Western blot using COMMD5 antibody. In the nucleus, the LPS group exhibited a significant decrease in COMMD5 protein expression level against the control group, while the TB-CM group showed a significant increase. The levels of protein expression in the cytoplasm did not show any significant variation across all the groups (Fig. 5c). Consequently, we confirmed that the expression levels of COMMD5 were high in the cell nucleus and summarized that COMMD5 would inactivate the pathway related to NF-κB, a mechanism related to the inflammatory response. Therefore, mRNA and protein expression were checked to test NF-κB activation. *NF-κB* mRNA expression levels were decreased in a TB-CM concentration-dependent manner in LPS-induced inflammation in HMEECs (Fig. 5d). In the cell nucleus, phospho-NF-κB (active form, Ser468), NF-κB, and β-actin antibodies were used to check for the presence of a band at about 65 kDa on an SDS–polyacrylamide gel, and the TB-CM group (20%), Phospho-NF-ĸB activity was significantly decreased (Fig. 5e). These results suggest that TB-CM can increase COMMD expression and reduce inflammation by participating in the NF-κB pathway.

### COMMD5 knockdown disrupts the anti-inflammatory response by TB-CM in LPS-induced HMEECs

**Figure 6:**
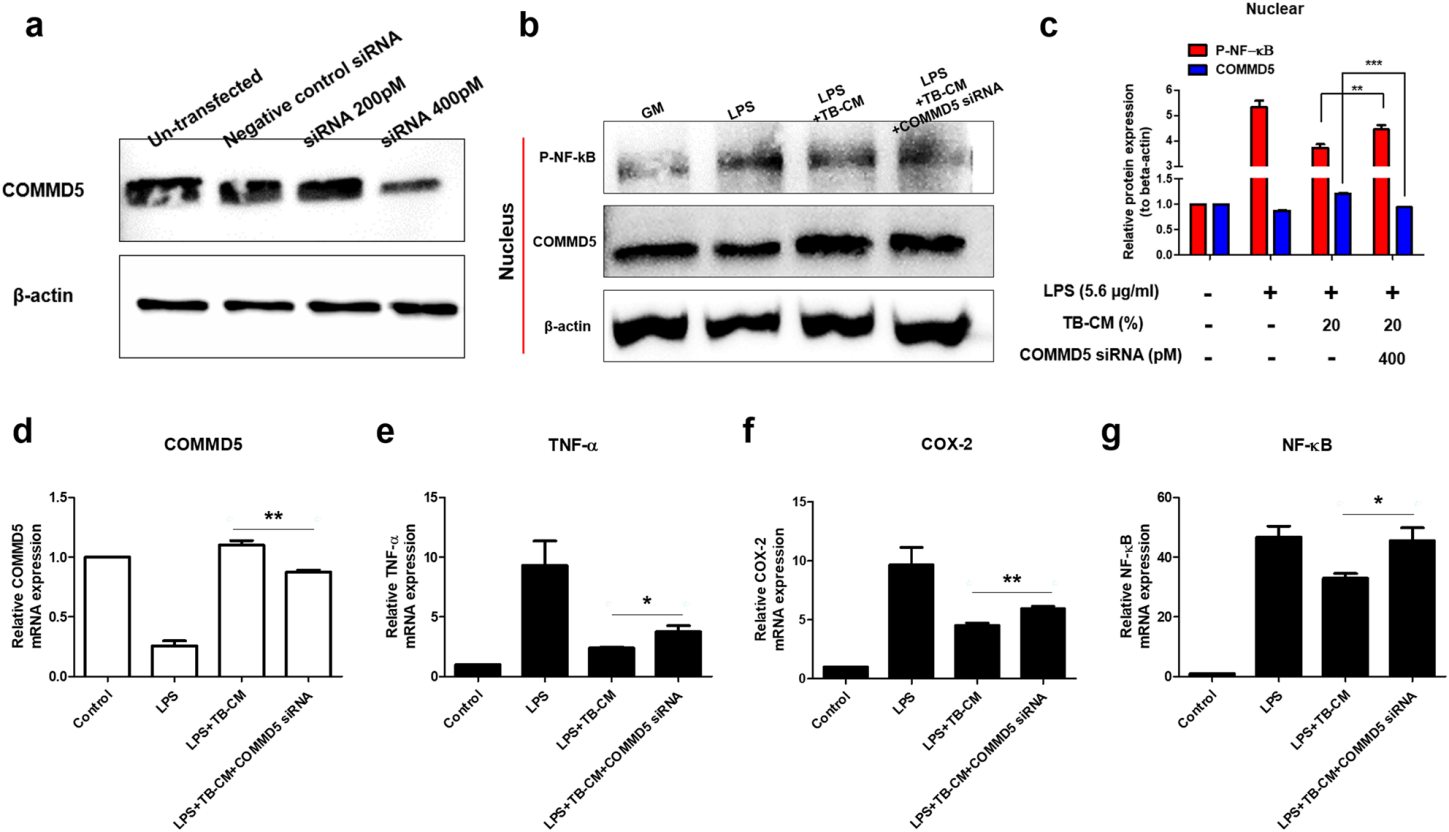
Copper metabolism MURR1 domain (COMMD5) downregulation promotes an inflammatory response in lipopolysaccharide (LPS)-induced human middle ear epithelial cells (HMEECs). (**a**) HMEECs were subjected to transfection with siRNA targeting COMMD5 and β-actin, followed by assessment of protein levels using immunoblot analysis. The COMMD5 protein expression in the cells was significantly reduced, indicating that the COMMD5 protein was effectively knocked down. (**b**) Western blot assay and (**c**) quantified data show the expression levels of COMMD5, and Phospho-nuclear factor (NF)-ҡB. When COMMD5 knockdown LPS-induced HMEECs were treated with TB-CM, the protein expression levels of pNF-ҡB were significantly increased. COMMD5 was significantly decreased. The mRNA expression levels of (**d**) *COMMD5*, (**e**) *TNF-α*, (**f**) *COX-2*, and (**g**) *NF-ҡB*. Inflammatory markers were increased in the TB-CM group when COMMD5 siRNA was transfected. All experiments were performed separately on a minimum of three occasions. *P < 0.05, **P < 0.01, and ***P < 0.001.

To confirm whether the upregulation of COMMD5 by TB-CM mediated anti-inflammation, knockdown was performed by COMMD5 siRNA transfection in HMEECs. In total cells, the protein level of COMMD5 decreased with increasing siRNA concentration (Fig. 6a). In the protein expression of pNF-κB, NF-κB, COMMD5, and β-actin extracted from cell nuclei from HMEECs, pNF-κB was significantly increased in the COMMD5 knockdown group against the TB-CM group. Levels of COMMD5 were increased in the TB-CM group against the control group but significantly decreased in the COMMD5 siRNA group (Fig. 6b,c). Results suggested that upregulation of COMMD5 by TB-CM reduced NF-κB activity, an inflammation-related mechanism. To evaluate the results of TB-CM-mediated siRNA COMMD5 downregulation in HMEECs cells, we performed RT-qPCR. Treatment with TB-CM increased the expression of *COMMD5* mRNA, but knockdown of COMMD5 did not increase mRNA expression level with treatment with TB-CM (Fig. 5d). Inflammation markers (TNF-α, COX-2, and NF-κB) were significantly increased in the siRNA group than in the TB-CM group (Fig. 6e–g). Knockdown of COMMD5 suggests involvement in TB-CM-induced anti-inflammation in LPS-induced HMEECs.

### TB-EVs reduced inflammatory cytokines caused by LPS in HMEECs

**Figure 7:**
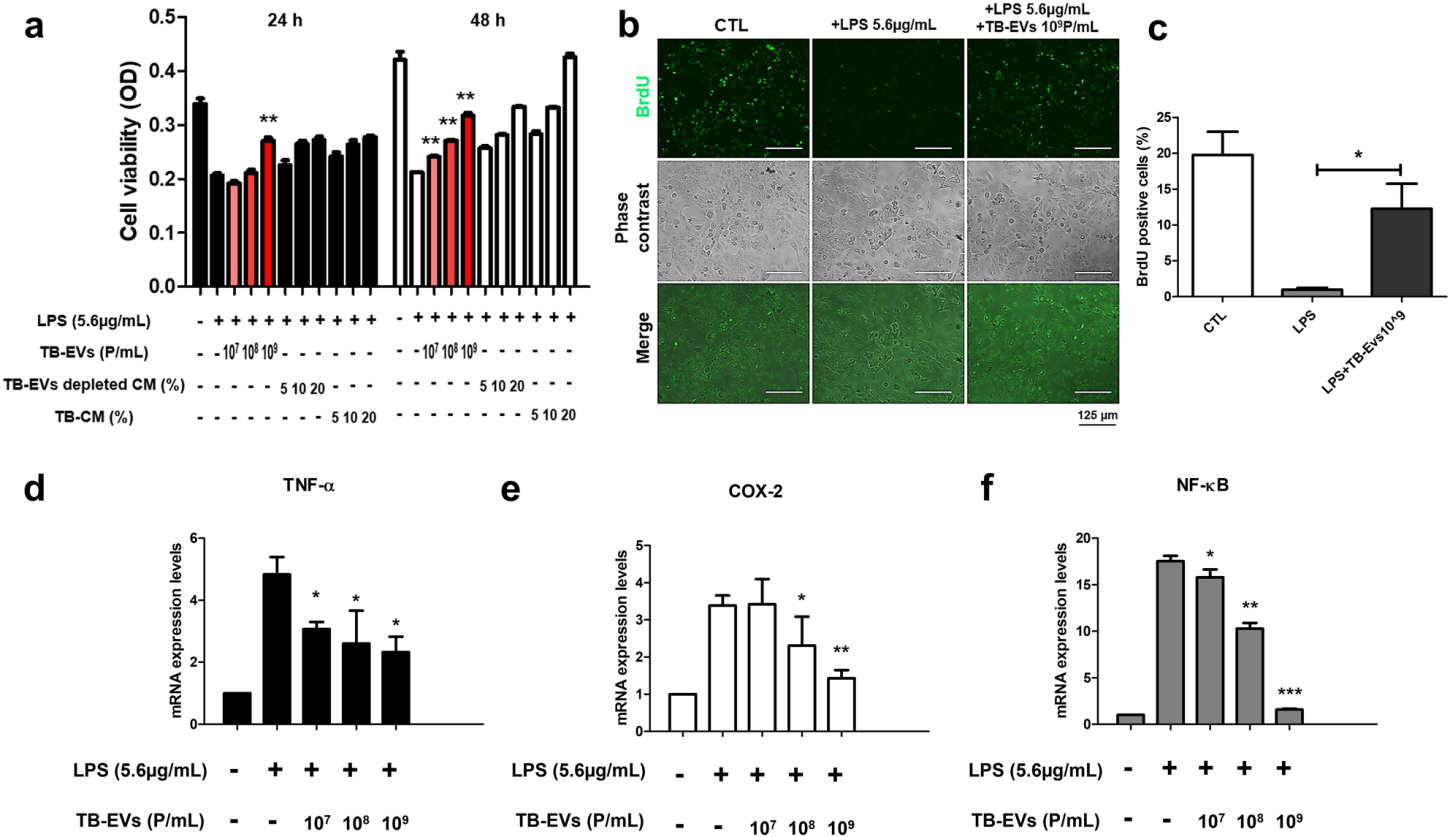
Trophoblast-derived extracellular vesicles (TB-EVs) increased cell viability and decreased mRNA expression levels of inflammatory markers in lipopolysaccharide (LPS)-treated human middle ear epithelial cells (HMEECs). (**a**) TB-EVs (1 × 10^7^, 1 × 10^8^, and 1 × 10^9^ particles/ml), trophoblast-conditioned medium (TB-CM) (5, 10, and 20%), and TB-EVs depleted TB-CM (5, 10, and 20%) were evaluated for cell viability of LPS-induced HMEECs for 24 h and 48 h. Concentration-dependent effects of TB-EVs on cell viability were increased in LPS-induced HMEECs. (**b**) Immunofluorescence analysis of BrdU incorporation (green) was performed in the existent or non-existent TB-EVs in LPS-induced HMEECs. The expression level of BrdU-positive cells decreased in LPS-induced cells compared to the control group and increased in the TB-EVs group. Scale bar 125 μm. (**c**) The BrdU positivity rate was examined for comparison. In LPS-induced HMEECs, the mRNA expression levels of (**d**) *TNF-α*, (**e**) *COX-2*, and (**f**) *NF-ҡB* decreased in a TB-EVs dose-dependent manner (10^7^, 10^8^, and 10^9^ p/ml). *P < 0.05, **P < 0.01, and **P < 0.001.

We hypothesized that TB-derived EVs from TB-CM treatment would also have anti-inflammatory factors and to check this, TB-EVs were extracted from TB-CM and exosomes were characterized. As a result of analyzing the shape and size of EVs with nanoparticle tracking analysis (NTA), the size of TB-EVs was 174.4 ± 3.2 (Fig. S2a). As a result of FACS analysis of the exosome surface marker CD63, the CD marker of TB-EVs was analyzed at 89.9% (Fig. S2b). Protein expression levels for exosome markers were analyzed by Western blot and showed high expression in TB-EVs (Fig. S2c). For the anti-inflammatory impact of TB-EVs in HMEECs induced by inflammation, TB-EVs were prepared by mixing with stem cell basal culture medium in a concentration-dependent method (1 × 10^7^, 1 × 10^8^, and 1 × 10^9^ particles/mL). LPS and TB- EVs were treated, cultured for 24 h or 48 h, and cell viability was evaluated with CCK-8. In both 24 h and 48 h, it was observed that cell viability increased in a concentration-dependent manner in each group. The concentration-dependent increase in cell viability in the TB-EVs group suggests the anti-inflammatory potential of TB-EVs. At 24 h, cell viability was similar among 10^9^ P/mL TB-EVs (0.270 ± 0.008), 20% TB-EVs-depleted CM (0.272 ± 0.009), and 20% TB-CM (0.2778 ± 0.003). However, at 48 h, 10^^9^ P/mL TB-EVs (0.317 ± 0.006), 20% TB-EVs-depleted CM (0.334 ± 0.001), and 20% TB-CM (0.425 ± 0.010) exhibited the highest cell viability in TB-CM, with TB-EVs-depleted CM and TB-EVs following in order. These results suggest that TB-EVs may have potential as anti-inflammatory agents in LPS-induced HMEECs and speculate that TB-CM contains more abundant anti-inflammatory components compared to TB-EVs (Fig. 7a). The expression level of BrdU-positive cells decreased in LPS-induced cells compared to the control group and increased in the TB-EVs group (Fig. 7b). The percentage of BrdU-positive cells was measured in the control group (19.784 ± 4.558), LPS group (1.241 ± 0.345), and TB-CM group (13.949 ± 3.95). TB-EVs group demonstrated a significant increase in BrdU-positive cells when compared to the LPS group (Fig. 7c). The anti-inflammatory efficacy of TB-EVs was evaluated using inflammatory gene markers. *TNF-α*, *COX-2*, and *NF-ĸB* were each decreased in a TB-EVs concentration-dependent manner (Fig. 7d–f).

## Discussion

TBs deliver nutrients and remove waste products necessary for embryo maturation and development during pregnancy between the mother and the fetus and are involved in metabolism related to the synthesis or secretion of various substances within the placenta^31^. In addition, placenta growth factor expression has been reported in TBs^21^. Cytotrophoblast is widely known to inhibit NF-ĸB activity, MMP9, and TNF-α production of macrophages stimulated by TLR or *Streptococcus*^22^.

We hypothesized that TB-derived CM or exosomes might affect the inflammatory effects of otitis media. In this study, an in vitro otitis media model (Fig. S1) was presented in HMEECs with LPS, an inflammation-inducing substance, and the anti-inflammatory effect was demonstrated as the concentration of TB-CM and TB-EVs increased. The HMEEC viability decreased to < 55% after 24 h of exposure to 5.67 ± 0.25 µg/mL LPS, as confirmed by BrdU and CCK-8 assays. We discovered that exposure to LPS for 24 h induced apoptosis, whereas simultaneous exposure to LPS and TB-CM reduced apoptosis (Fig. 1a–c). TNF-α is a cytokine that is involved in inflammatory and immune responses, and its expression is increased in otitis media^32,33^. Furthermore, it induces apoptosis or produces IL-1 and IL-6 to appear in various human diseases, such as inflammatory diseases and cancer^33^. The *COX-2* gene mediates inflammation and induces prostaglandin H_2_ production^34^. We further discovered that the TNF-α and COX-2 expression levels were significantly increased upon induction of inflammation and decreased by TB-CM (Fig. 1d,e). The discovery of cell-derived anti-inflammatory factors will prove valuable for the development of new materials by reducing cell death and inflammatory factors in the middle ear. Anti-inflammatory effects of TB-CM were found in cells induced by inflammation and this effect was mediated by otitis media-related osmotic control channels and mucin genes (Figs. 2 and 3).

RNA-sequencing was performed to analyze the DEGs of the LPS and LPS + TB-CM groups in LPS inflammation-induced cells (Fig. 4). We propose the pathway of the gene *adenosine A2a receptor (ADORA2A)*, which was found to be upregulated in the DEG analysis, as one of the pathways that reduce the inflammatory response by TB-CM in LPS-induced HMEECs. In the comparison between the LPS group and the TB-CM group, ADORA2A exhibited a 28.061-fold upregulation in RNA sequence analysis. Additionally, mRNA expression levels measured using qRT-PCR showed a 22.703-fold increase between the two groups. Therefore, the increased expression of ADORA2A was confirmed not only in the discussed RNA sequence analysis but also in qRT-PCR (Fig. S3). In the GO term analysis (BP results), genes related to the G protein-coupled receptor signaling pathway were analyzed (Fig. 4d,e). ADORA2A receptor refers to the adenosine receptor. The role of the G protein-coupled adenosine receptor was confirmed^35^. One of its functions is to protect tissues from inflammation by suppressing immune cells^35^. In the ADORA2A receptor signaling pathway, the A2A receptor binds to the Gs protein. Activation of ADORA2A causes the accumulation of intracellular levels of cAMP produced by the enzyme adenylyl cyclase. This causes protein kinase cAMP-dependent activation and activates cAMP-responsive element-binding protein, inhibiting NF-κB^36^. JNK and ERK pathways, which are MAPK signaling pathways, were also investigated^37^. In conclusion, the activation of these ADORA2A inhibits NF-κB activation and suppresses the inflammatory response.

**Figure 8:**
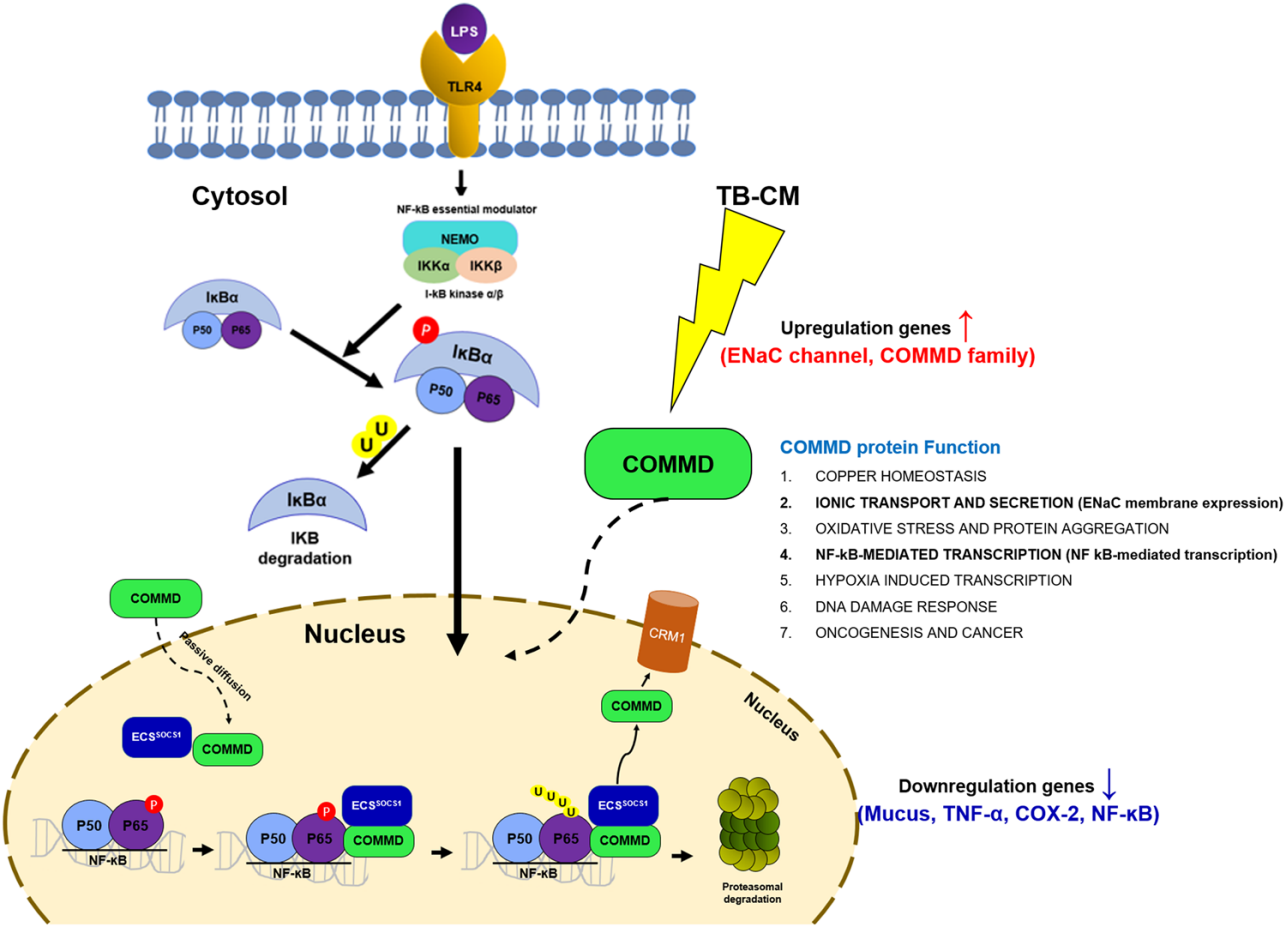
Schematic representation of the mechanism for trophoblast-conditioned medium (TB-CM)-induced anti-inflammatory in lipopolysaccharide (LPS)-induced human middle ear epithelial cells (HMEECs). *COMMD* copper metabolism MURR1 domain.

Another signaling pathway is presented by the COMMD protein. COMMD proteins are numbered 1–10 and refer to 50–65 amino acids with high homology inside the carboxy-terminal region designated as the COMM domain. The N-terminal region is uniquely present in every COMMD protein, COMMD is numbered according to this region^38^. Furthermore, in terms of the functional aspects of COMMD1 − 10, there is no clear consensus regarding their specific functions^38^. These proteins are known to interact with each other when assessed in a PPI network^38,40^. Therefore, COMMD5, which showed the highest mRNA expression level in qRT-PCR analysis, was selected. To confirm the upregulation of the COMMD protein upon LPS-induced HMEECs exposure to TB-CM, we assessed the COMMD gene and protein expression levels (Fig. 5), as well as knockdown COMMD5, which exhibited the highest expression level. We verified that inflammatory factors were upregulated (Fig. 6). In addition, we hypothesized that there would be anti-inflammatory factors in TB-EVs derived from TB-CM and extracted TB-EVs from TB-CM, confirmed the characteristics of EVs (Fig. S2), and confirmed the anti-inflammatory effect of TB-EVs (Fig. 7). The regulation of COMMD proteins in NF-κB signaling and their significant involvement in protein interactions have been examined^39^. COMMD passes through nuclear pores via passive diffusion and binds with the Ubiquitin E3 ligase complex, ECS^SOCS1^, inside the nucleus. The Ser468 residue phosphorylation advances the association of P65 and COMMD1-ECS^SOCS1^. COMMD1 acts as a hub to promote interaction with ECS^SOCS1^, resulting in ubiquitination and subsequent proteasomal degradation of P65^40,41^. Therefore, we hypothesized COMMD proteins could be involved in the NF-ĸB pathway (Fig. 8).

In this study, we aimed to investigate the therapeutic potential of TB-CM and TB-EVs in addressing middle ear inflammation, with the objective of overcoming the limitations associated with conventional long-term drug treatments and surgical interventions. EVs, which are small structures produced within cells and released externally, play a vital role in facilitating cell-to-cell communication and signal transmission^42^. Recent studies have raised increasing interest in the possibility that stem cell derived EVs may have important effects on inflammation and immune responses^43^. EVs have the capacity to modulate interactions between immune cells and contribute to the regulation of immune responses, particularly by suppressing or regulating inflammatory reactions^44^. Moreover, they facilitate the exchange of information and communication among immune cells, which is imperative for the regulation of immune responses and the control of immune cell activities. However, the study of the anti-inflammatory properties of TB-EVs, which are components of placental tissue, has been somewhat limited. Our research emphasizes the potential of TB-CM and TB-EVs in the treatment of middle ear inflammation. Nonetheless, the application of in vitro models presents certain constraints, and we anticipate potential limitations in clinical implementation. Consequently, further research involving in vivo models will be essential in the future. Through this study, we have advanced our understanding of the potential application of new anti-inflammatory agents, taking into consideration various patient conditions, and have gained insight into how EVs contribute to the regulation of inflammation and the immune system. This research has the potential to open new avenues for the treatment and prevention of inflammatory and immune-related diseases in the future.

## Materials and methods

### Cell culture

HMEEC (cells into which human papillomavirus E6/E7 genes were inserted), were provided by Dr. David at the House Ear Institute in the USA. Cells were cultured in an airway epithelial cell growth medium (C-21160; PromoCell, Heidelberg, Germany) and supplement (C-39165; PromoCell, Heidelberg, Germany). HTR-8/SVneo was purchased from ATCC (CRL-3271; VA, USA) and cultured in RPMI-1640 (11875-093; Gibco, Paisley, UK) with 1% penicillin/streptomycin (P/S) (15140-122; Gibco, Grand Island, USA) and 5% fetal bovine serum (FBS) (16000-044; Gibco, Grand Island, USA). All cells were cultured in humidified air in a 37 °C incubator, containing 5% CO_2_.

### Preparation of TB-CM and TB-EVs

To obtain TB-CM, HTR-8/SVneo cells were rinsed twice with phosphate-buffered saline (PBS) (10010; Gibco, NY, USA) upon 60% confluence. The medium was replaced with RPMI-1640 medium (Gibco) with 5% exosome-depleted FBS (Gibco) and 1% P/S (Gibco). CM was incubated for 48 h and collected in a 50 mL tube. CM was obtained by centrifugation (Union 32R Plus; Hanil Science, Inchon, Korea) at 300×*g* for 10 min to isolate cellular waste, followed by filtration with a syringe filter (pore size 0.22 µm) (CLS431229; Corning, NY, Germany). To isolate TB-EVs from the supernatant following the removal of cellular debris in TB-CM, we used an ultracentrifuge (Optimal Max-Xp, Beckman Coulter, CA, USA). The supernatant was first centrifuged at 300×*g* for 10 min at 4 °C, and the upper layer was collected. Subsequently, another centrifugation was performed at 3000×*g* for 10 min at 4 °C, and the upper layer was collected. Finally, the pellet was obtained by centrifuging at 100,000×*g* for 150 min at 4 °C and then diluted in filtered PBS for recovery.

### Cell counting Kit-8 assay (CCK-8 assay)

HMEEC viability was assessed using a CCK-8 assay (CK04; Dojindo, Kumamoto, Japan). HMEECs were inoculated at a density of 1 × 10^4^ cells per well in a 96-well dish and incubated for 24 h. Cells were simultaneously treated with LPS (5.6 µg/mL; Sigma, St. Louis, MO, USA) and TB-CM (0, 10, 15, 20, 25, and 30%), with a control group without LPS and TB-CM addition. After incubation at 37 °C for 24 h or 48 h, a total volume of 10% CCK-8 solution was added to each well. The cells were then incubated at 37 °C for 1 h. Subsequently, the optical density was gauged at a wavelength of 450 nm with the aid of a microplate reader (SpectraMax 190 Microplate Reader, California, USA).

### BrdU assay

Cell proliferation was assessed using BrdU immunocytochemistry. HMEECs were cultured in 24-well plates with 25 µM BrdU (B5002; Sigma, St. Louis, USA), TB-CM, TB-EVs, and LPS. After 24 h, the cells were rinsed with PBS and subsequently immobilized with 4% paraformaldehyde (PFA) at 4 °C for 20 min. For DNA denaturation, the cells were incubated with 1N HCl at 4 °C for 10 min, 2N HCl at 17 °C for 10 min, and 2N HCl at 37 °C for 20 min. The cells were cultured with 0.1% Triton X-100 and 3% BSA in PBS at 37 °C for an hour. BrdU monoclonal antibody Alexa fluor 488 (1:1000, B35130; Invitrogen) was incubated at 4 °C for 24 h. After washing, it was observed with an automated fluorescence microscope (Evos FL Auto 2; Invitrogen, WA, USA).

### Quantitative real-time PCR

TRIzol™ reagent (15596018; Invitrogen, CA, USA) was applied to extract total RNA from every set of HMEECs. Add 1 ml of TRIzol™ reagent to each group to lyse the cells. Add 200ul of chloroform (C2432-500ML, Sigma, USA), mix well, incubate for 3 min, and then centrifuge at 19,000×*g* for 20 min. Collect only the supernatant, add 2-propanol (19516-500ML, Sigma, Germany) in a 1:1 ratio, and gently invert. Centrifuge at 19,000×*g* for 20 min. Completely remove the supernatant, perform two washes with 70% ethanol, discard the supernatant. Dissolve the pellet in sterilized distilled water, measure the RNA concentration using a nanodrop (AZY1707715, Thermo Scientific, USA). To synthesize cDNA, 1 μg of RNA was subjected to reverse transcription using a PrimeScript™ 1st strand cDNA synthesis kit (6210A; Takara, Kusatsu, Shiga). The RT-qPCR mixture constituted 1 µl cDNA, 5 pmol of forward and reverse primers, and 10 µl of SYBR green Master Mix (Life Technology, Warrington, UK), for a total volume of 20 µl. RT-qPCR was completed using the Quant Studio 6 Flex system. The denaturation process was performed at 95 °C for a duration of 15 s, followed by annealing at 60 °C for a period of 1 min, and 40 cycles were repeated. All experiments were run in triplicate. The Ct value was calculated using a relative quantification method by normalizing the mRNA expression levels of GAPDH. The primer sequences data used for RT-qPCR are listed in Tables S1 and S2.

### Western blot analysis

Thermo Scientific's NE-PER™ reagents (78,833; Thermo, Rockford, USA) were used to extract nuclear and cytoplasmic proteins from HMEECs. The PER™ reagents was used according to the manual^5^. The total protein was incubated for 1 h at 4 °C in RIPA buffer. Cells were lysed and quantified with the use of a bicinchoninic acid (BCA) assay to achieve the same quantities of protein. TNF-α (1:1000, SC-52746; Santa Cruz), COX-2 (1:1000, ab52237; Abcam), ALIX (1:1000, EXOAB-ALIX-1; SBI), CD63 (1:1000, EXOAB-CD63-1; SBI), CD81 (1:1000, EXOAB-CD81A-1; SBI), COMMD5 (1:1000, 10393-1-AP; Proteintech), NF-κB (1:1000, SC-8008; Santa Cruz), Phospo-NF-κB (1:1000, SC-136548; Santa Cruz), and β-actin (1:1000, 8H10D10; Cell Signaling) were used as antibodies and incubated for a day at 4 °C. Anti-Mouse IgG-HRP (1:4000, 7076S; Cell signaling) and anti-Rabbit IgG-HRP (1:4000, 7074S; Cell Signaling) was added for 2 h at room temperature. Bands were visualized using chemiluminescence (SuperSignal™ West Femto Maximum Sensitivity substrate; Meridian Rd., Rockford, USA). Chemiluminescence analyzer system was used to visualize the bands, which was done within 10 min.

### Immunofluorescence staining

HMEECs were seeded at 2 × 10^5^ cells/well on a cell culture slide (8 wells; SPL Life Sciences) and incubated overnight. The cells were rinsed twice with PBS and induced with LPS (56 µg/mL) and TB-CM (10% and 20%) in the growth medium. Controls were not treated with LPS or TB-CM. Cells were cultured for 24 h, washed twice with PBS, and fixed with 4% PFA for 15 min. The cells were then incubated in 0.3% Triton X-100 (in PBS) as a permeabilization solution at 37 °C for 5 min. BSA (3% in PBS) was added to the permeation solution and incubated for blocking for 1 h at 37 °C. The primary antibody, MUC5AC (1:500, MA1-38,223; Thermo Fisher) was diluted in blocking buffer and incubated at 4 °C for 24 h. After washing thrice for 5 min, Alexa Fluor™ 488 goat anti-mouse IgG (H + L) (1:2000, A11001; Invitrogen) was incubated for 2 h at 37 °C in the dark. They were washed thrice for 5 min with PBS, and stained nuclei with DAPI. Cellular fluorescence signals were observed with a confocal microscope (Zeiss LSM700, Oberkochen, Germany) and images were analyzed with ZEN lite (version 2.3.)

### RNA-sequencing

To identify DEG, we conducted RNA sequencing. In the library preparation phase, we isolated total RNA from LPS-induced HMEECs and LPS-induced HMEECs treated with TB-CM (20%) for 24 h. We constructed transcriptome libraries following the manufacturer's protocol using the TruSeq Stranded mRNA Reference Guide (Illumina, San Diego, CA, USA)^46^. We generated cDNA from fragmented RNA through reverse transcription, attaching different adapters to both ends of the cDNA fragments and ligating them. After PCR amplification to obtain an insert size of 200–400 bp, we performed size selection to ensure the quality of the reads obtained through sequencing. We conducted an overall read quality analysis, including total bases, total reads, and GC (%) statistics. To reduce bias in the analysis results, we underwent a preprocessing step to remove artifacts such as low-quality sequences, adapter sequences, contaminant DNA, and PCR duplicates. Preprocessed reads were then mapped to the reference genome using the HISAT2 program (v2.1.0). Using information from the reference-based aligned reads, we extracted expression profiles in terms of FPKM (Fragments Per Kilobase of transcript per Million mapped reads)/RPKM (Reads per Kilobase of transcript per Million mapped reads) values and TPM (Transcripts Per Kilobase Million) values, considering transcript length and depth of coverage^47^. We selected genes or transcripts with statistically significant differential expression values among more than two groups with different conditions using statistical hypothesis testing. To identify genes differentially expressed between the two groups, we extracted genes that satisfied the condition |fc|> = 2 and exactTest raw p-value < 0.05 using edgeR for the comparison groups (LPS and LPS + TB-CM). For DEGs with known gene information, we performed functional annotation and gene set enrichment analysis based on GO and KEGG pathways (https://www.genome.jp/kegg/pathway.html). GO analysis was conducted from three perspectives using the DAVID website (https://david.ncifcrf.gov/tools.jsp). To examine gene networks, we utilized Cytoscape (v3.9.1) tools and the stringAPP for analysis.

### siRNA transfection

siRNA sequences were designed for COMMD5 (5'-GUU UUA GGU UGG GCA UUU U-3', 5'-AAA AUG CCC AAC CUA AAA C-3') (Bioneer, Daejeon, South Korea). HMEECs were cultured in 12-well plates for one day at a cell confluency of 60–70%. Four hours before transfection, plates were washed twice with PBS and replaced with basal culture medium without added P/S and FBS. The transfected cocktail was prepared with 400 pmol of siRNA and 8 µl of lipofectamine in DMEM/F12 medium using Lipofectamine RNAiMAX (Invitrogen, Van Allen Way Carlsbad, CA, USA). Cells were then incubated for 15 min at room temperature to mix the complexes and 200 μl of transfection cocktail was added to each well. After 24 h, cells were harvested for Western blot assay and RT-qPCR analysis.

### NTA

EV size, concentration, and distribution were confirmed using NTA. For particle light scattering recordings, we utilized NTA equipment (Malvern, NS300, Grove Wood Road, UK), with a scientific CMOS (sCMOS) camera type and a red laser type. The samples were prepared by diluting TB-EVs (1 mL) at a ratio of 1:20. The laser irradiated the sample, while the camera captured the movement of particles. Three videos, recorded for 60 s each, were analyzed to visualize EV concentration, size, and distribution.

### Flow cytometry

To characterize microvesicles, microvesicles isolated from TB-CM were used for flow cytometry. Since exosomes are too small to be directly used for flow cytometry, exosomes were captured and analyzed with a bead with a diameter of 9.1 μm. Exosome cell surface protein markers CD63 (Exo-Flow Capture kit, EXOFLOW300A-1; SBI) were prepared according to the manufacturer’s instructions. Next, Exo-Flow FACS magnetic beads were prepared, and the bead-attached capture antibody was incubated at 4 °C for 24 h. Exosomes were then stained using Exo-FITC exosome satin, and cytometric data were measured using a Flow Cytometer Analyzer (BD LSRFortessa™ X-20 special order system, CA, USA).

### Statistical analysis

All statistics outcomes are expressed as averages of three or more experiments. To determine the importance among groups, the statistical evaluation software program PRISM 5.1 (San Diego, CA, USA) was used, and a two-way analysis of variance was performed. *P < 0.05, **P < 0.01, and ***P < 0.001 were considered statistically significant.

### Supplementary Information

Supplementary Information.

## Data Availability

The datasets generated and analyzed during the current study are available in the [PRJNA976048] repository, [https://www.ncbi.nlm.nih.gov/sra/PRJNA976048].
