# Supplementary material for: Inhibition of lipopolysaccharide-induced inflammation by trophoblast-conditioned medium and trophoblast-derived extracellular vesicles in human middle ear epithelial cells
Source: Sci Rep. 2023 Nov 14;13:19822. doi: 10.1038/s41598-023-46731-7 (PMC10645728; doi:10.1038/s41598-023-46731-7)
Supplement: Supplementary file 1 — Supplementary Information. [file 41598_2023_46731_MOESM1_ESM.pdf]

Supplement Tables

Table S1. ***COMMD*** **(1—10) Primer sequences used in quantitative RT-PCR.**

Primer	Direction	Sequence (5' to 3')
COMMD1	F	CGGAGCCAGCTATATCCAGA
	R	TTGGAAATGACAGCAGCTTG
COMMD2	F	CCAAGCCTTCCCAGTTATCA
	R	TGTTGAACCAAATGGAGCAG
COMMD3	F	AAAGCACCGAGCTGACAAGT
	R	CGCCAAGAAACATCCGTTAT
COMMD4	F	TGGCAGTGCTGAGTTTCATC
	R	ACCTGCCAACCTATTCATGC
COMMD5	F	TGCAACTCCATACCTGCATC
	R	GCTGCTGACCACAAACTTCA
COMMD6	F	AACTGGGTATGGCTGTGAGC
	R	AACTGCAGCAATTTCCTTGAA
COMMD7	F	GAGCAGCGAATTGGAGAAAG
	R	TCAGCAGAAACACTCCATGC
COMMD8	F	CAGGATTTTGATTGGCAGGT
	R	CTTATTCGCTGCTTCCAAGG
COMMD9	F	AAGGCCTCCTCGAAAGATGT
	R	AGAGGACAGGTCACGGAATG
COMMD10	F	ATTTCCACGGTTGCTCACTC
	R	AAAGCTGCTGGCTTCACATT

Table S2. Primer sequences used in quantitative RT-PCR.

Primer	Direction	sequence (5' to 3')
TNF-α	F	GAGGCCAAGCCCTGGTATG
	R	CGGGCCGATTGATCTCAGC
COX-2	F	CTGGCGCTCAGCCATACAG
	R	CGCACTTATACTGGTCAAATCCC
AQP4	F	TCTCATCTCCCTTTGCTTTG
	R	ACAGTCACTGCAGGGTTGAT
ENaC-α	F	GCAGTCCGATTTGTTCTGGT
	R	CAGGTGGACTGGAAGGACTG
ENaC-β	F	GACCAAAGCACCAATATCCC
	R	GAAGTAGATGTTGAGCTTGACAATTC
ENaC-γ	F	TCTACAACGCTGCCTACTCG
	R	TCCACCATCTTTGTCTGGAA
MUC1	F	CGCCGAAAGAACTACGGGCAGCTG
	R	CAAGTTGGCAGAAGTGGCTGCCAC
MUC2	F	ACCCGCACTATGTCACCTTC
	R	GGACAGGACACCTTGTCGTT
MUC5AC	F	GGTGACTTCGACACACTGGA
	R	TGGACCCTGATCTGGTAGTTG
NF-κB	F	GGGGCTATAATCCTGGACTCTT
	R	GGCGGATTAGCTCTTTTTCC
ADORA2A	F	AGGCAGCAAGAACCTTTCAA
	R	CTAAGGAGCTCCACGTCTGG
GAPDH	F	TCGCCCCACTTGATTTTGG
	R	GCAAATTCCATGGCACCGT

Supplementary Figure

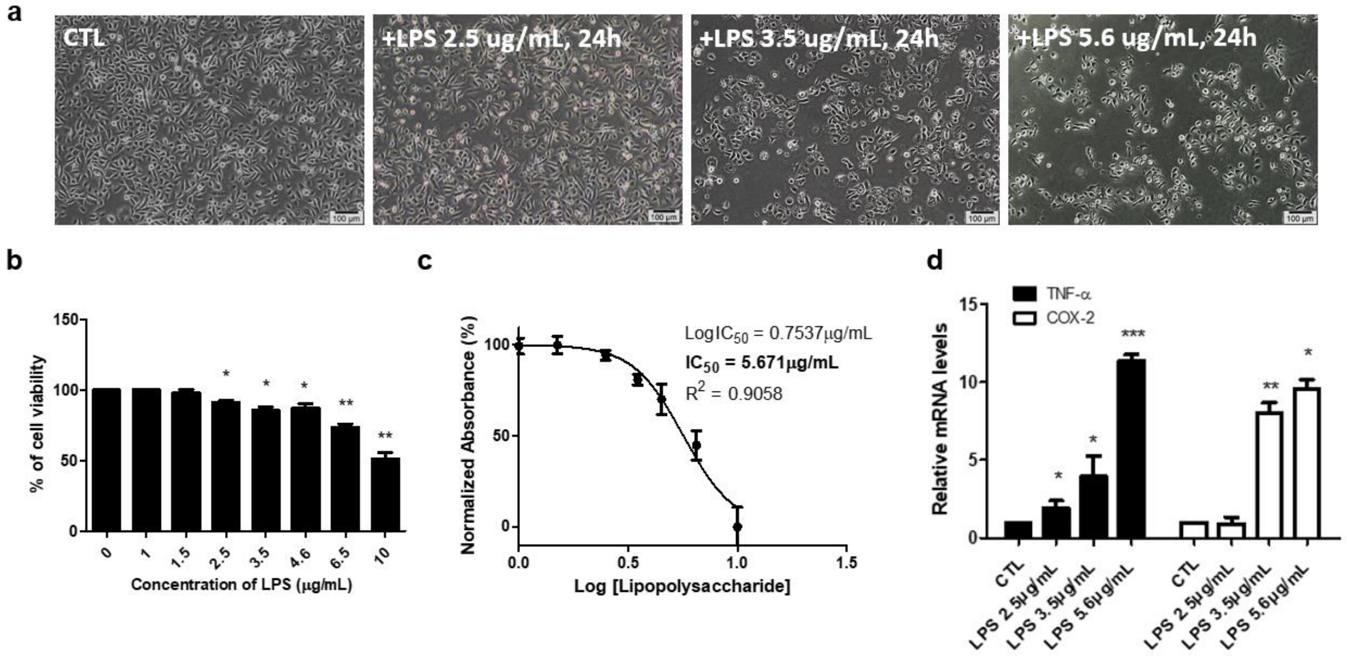

Supplement Figure S1. Cell viability and mRNA expression levels of *TNF-α* and *COX-2* in HMEECs treated with LPS. (a) Images of HMEECs after treatment with 0, 2.5, 3.5, and 5.6 μg/mL LPS for 24 h. The number of cells in the LPS-treated group was decreased compared to the control group. (b) Cell viability of HMEECs after treatment with 0, 1, 1.5, 2.5, 3.5, 4.6, 6.5, and 10 μg/mL for 24 h. Cell viability decreased in an LPS concentration-dependent manner. (c) IC_{50} (half maximal inhibitory concentration) values were obtained using GraphPad Prism 5.1 software and showed the LPS IC_{50} concentration (IC_{50} = 5.6 μg/mL) in HMEECs. The LPS-treated group exhibited 50% cell death compared to the control group. (d) HMEECs were treated with LPS (2.5−5.6 μg/mL) for 24 h. Quantitative real-time PCR was performed to evaluate the mRNA expression levels of *TNF-α* and *COX-2*, which are inflammatory cytokine genes. The mRNA expression level increased in an LPS concentration-dependent manner. P-values were indicated as $*P<0.5$, $**P<0.1$ and $***P<0.01$.

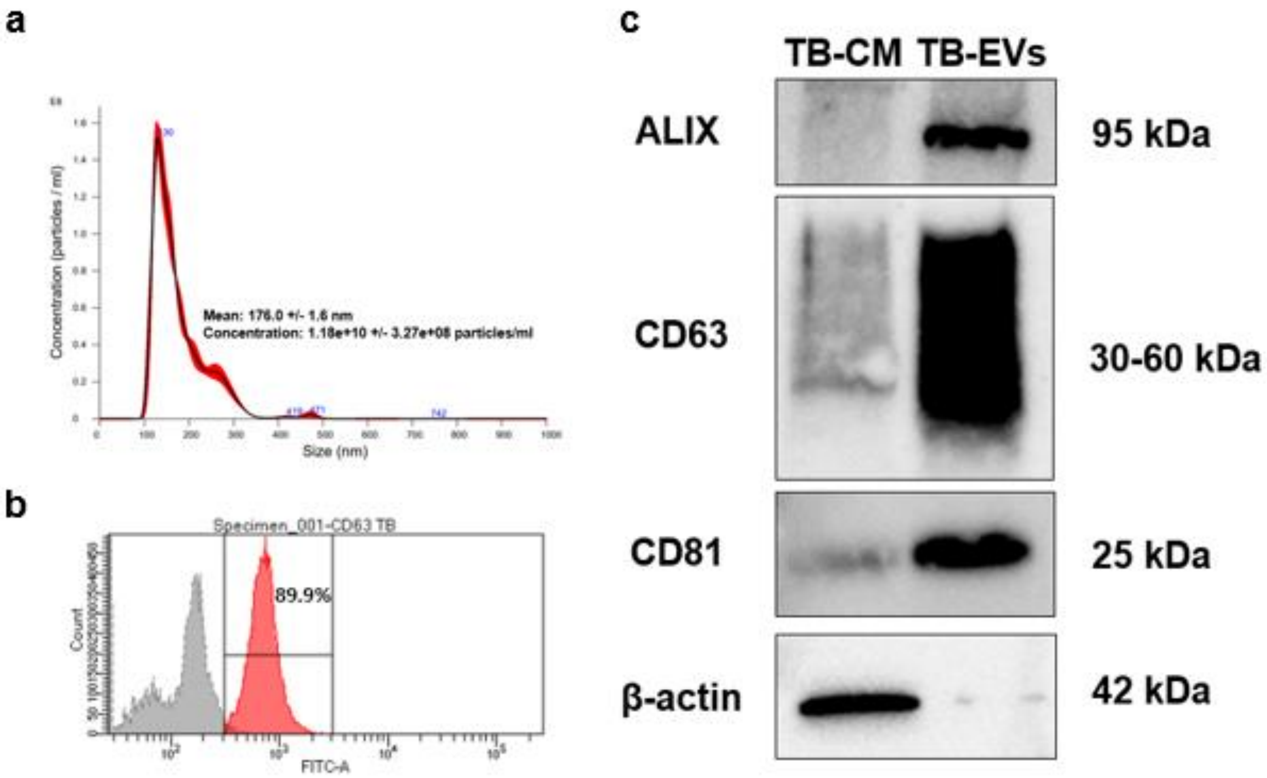

Supplement Figure S2. Trophoblast-Extracellular Vesicles (TB-EVs) characterization. (a) NTA analysis. (b) FACS analysis of the exosome surface marker CD63. (c) TB-CM and TB-EVs were identified by Western blotting with exosome markers ALIX, CD63, CD81, and β-actin.

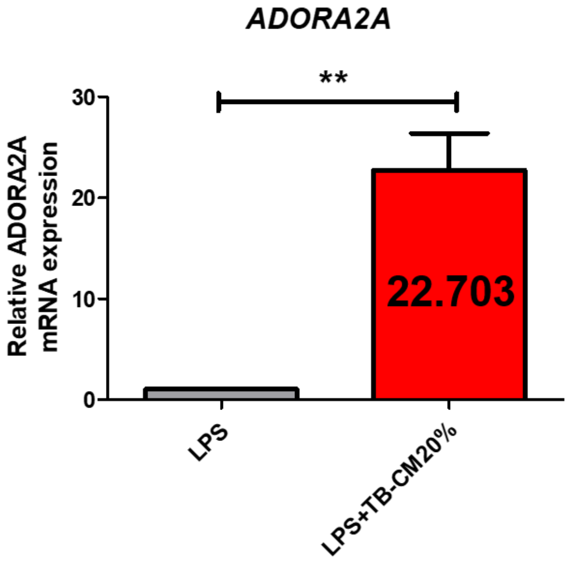

Supplement Figure S3. The upregulation of the gene *ADORA2A* in RNA sequencing was confirmed by Quantitative real-time PCR for mRNA expression levels. *ADORA2A* exhibited a 28.061-fold upregulation in RNA sequence analysis. As measured by qRT-PCR, the mRNA expression levels showed a significant 22.703-fold increase between the two groups. This increase in *ADORA2A* expression was confirmed in both RNA sequence analysis and qRT-PCR. P-values were indicated as **P<0.5, **P<0.1 and ***P<0.01.*

Supplement Figure S4. Western blot Raw data.

Fig. 1e

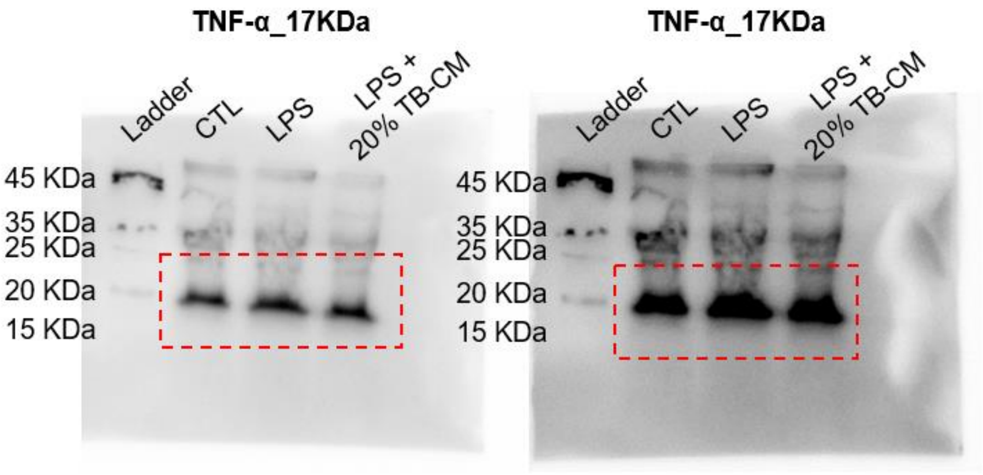

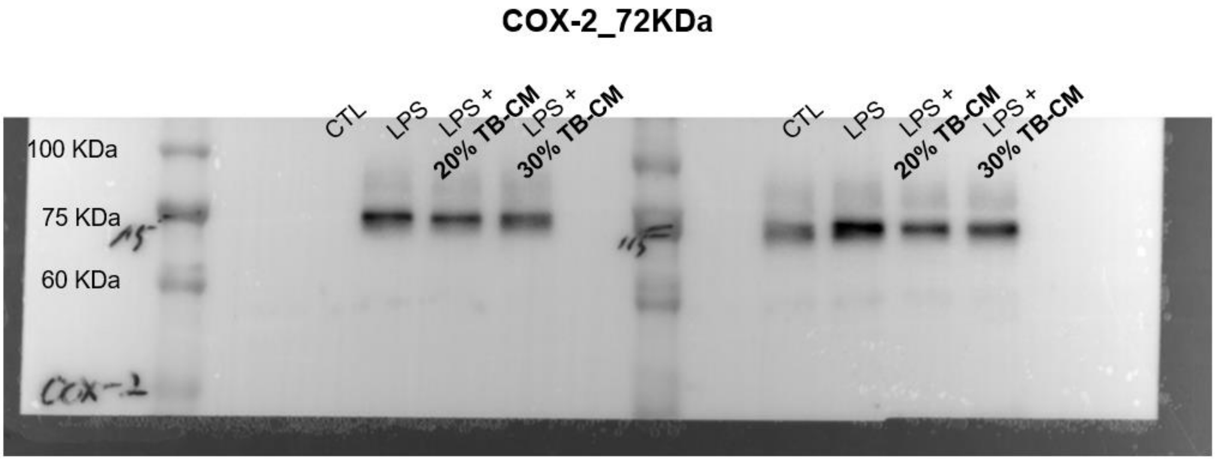

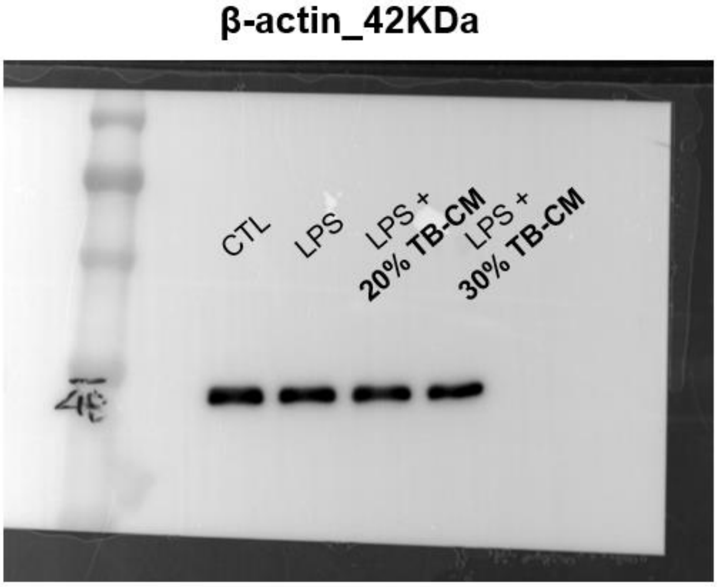

Fig. 5c

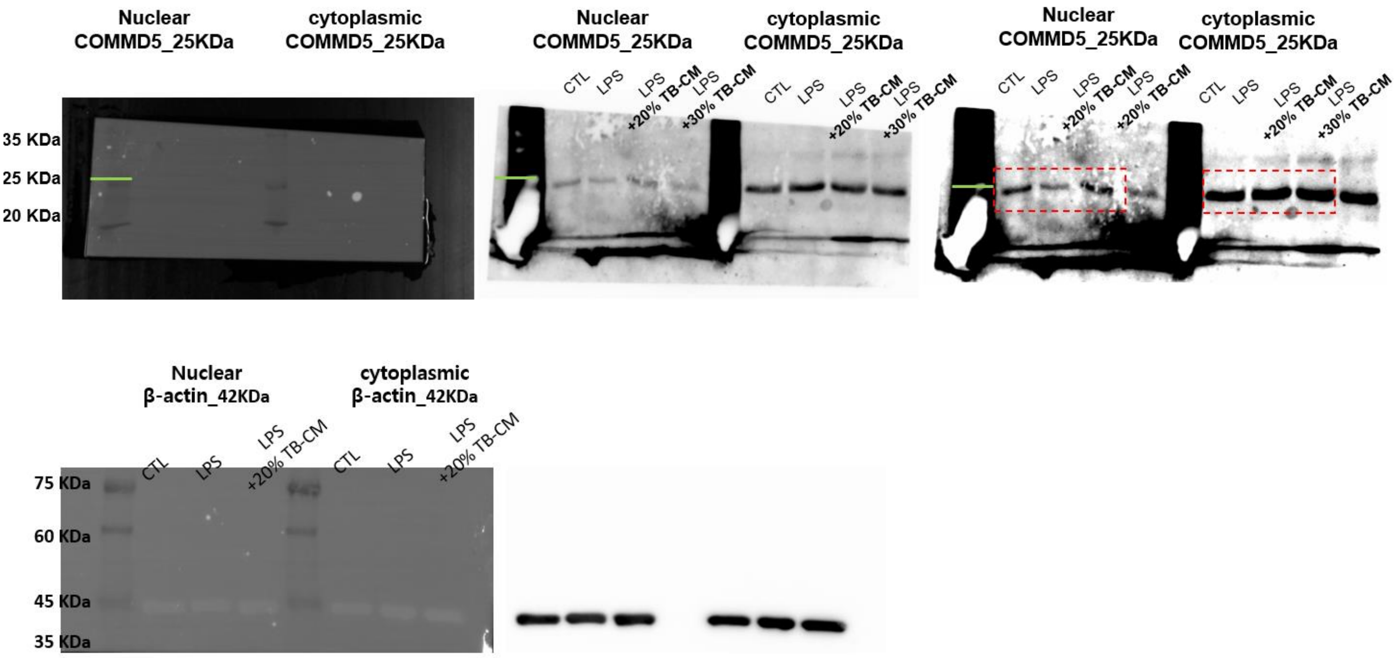
Nuclear
COMMD5_25KDa
cytoplasmic
COMMD5_25KDa
35 KDa
25 KDa
20 KDa
CTL
LPS
LPS +20% TB-CM
LPS +30% TB-CM
Nuclear
β-actin_42KDa
cytoplasmic
β-actin_42KDa
LPS +20% TB-CM
75 KDa
60 KDa
45 KDa
35 KDa

Fig. 5e

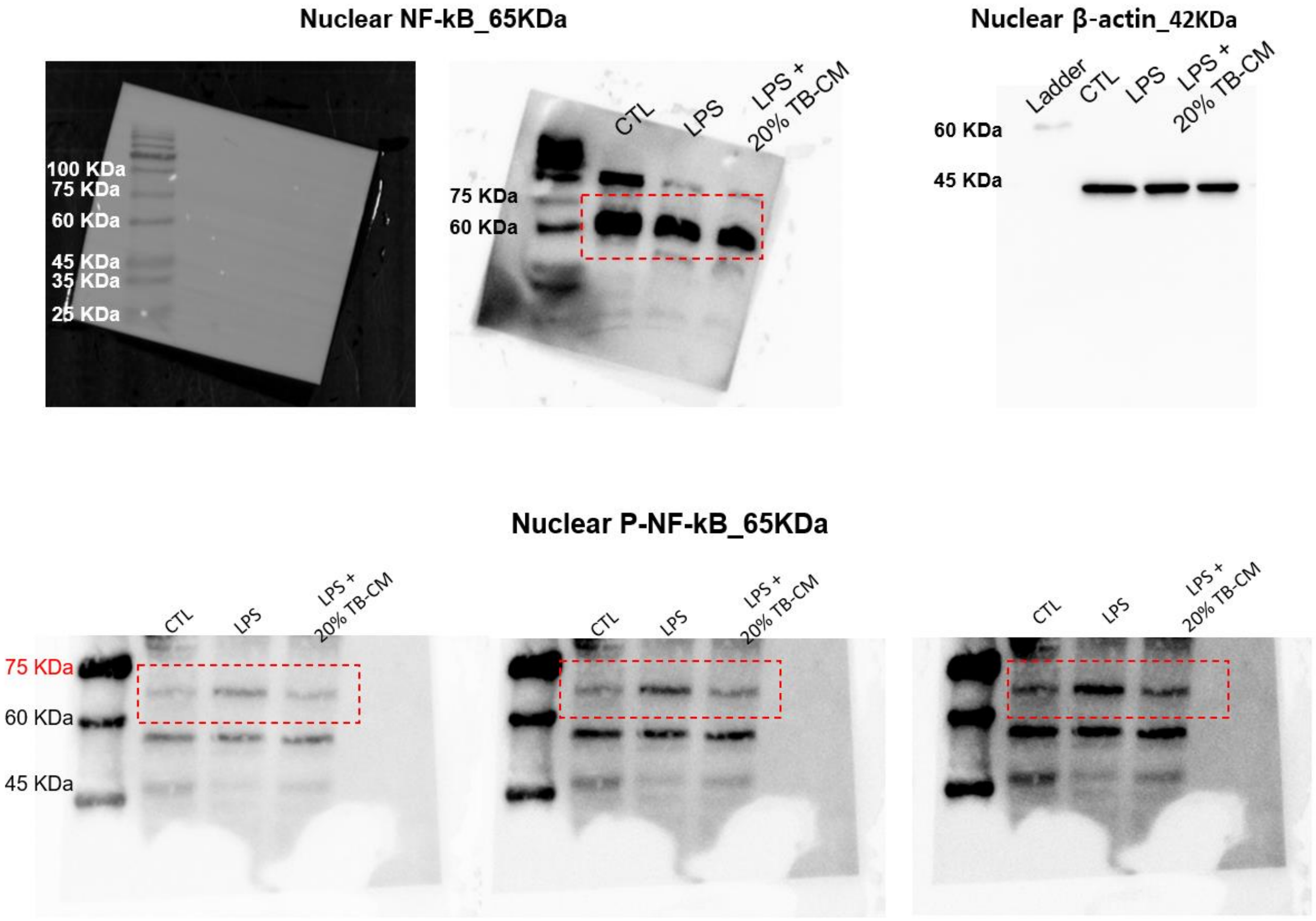
Nuclear NF-kB_65KDa
100 KDa
75 KDa
60 KDa
45 KDa
35 KDa
25 KDa
CTL
LPS
LPS + 20% TB-CM
Nuclear β-actin_42KDa
Ladder
60 KDa
45 KDa
Nuclear P-NF-kB_65KDa
75 KDa
60 KDa
45 KDa

Fig. 6a

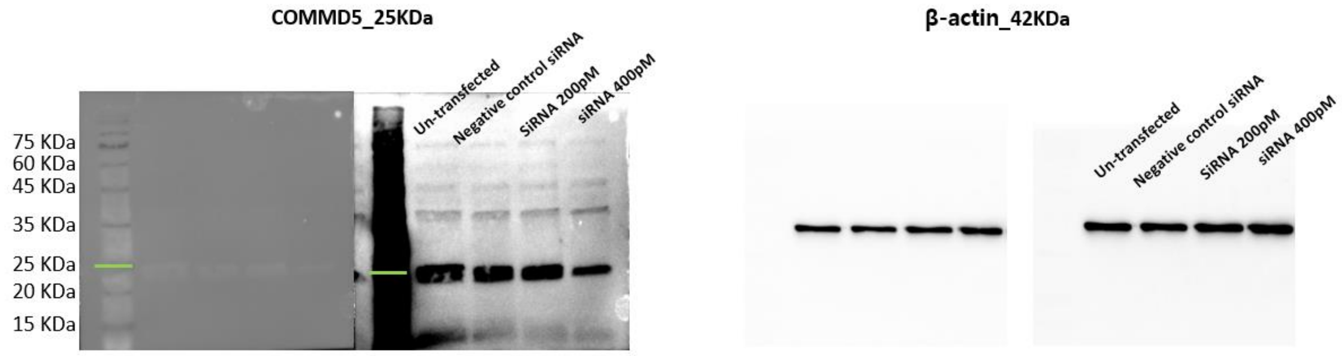
COMMD5_25KDa
β-actin_42KDa
75 KDa
60 KDa
45 KDa
35 KDa
25 KDa
20 KDa
15 KDa
Un-transfected
Negative control siRNA
SiRNA 200pM
siRNA 400pM

Fig. 6b

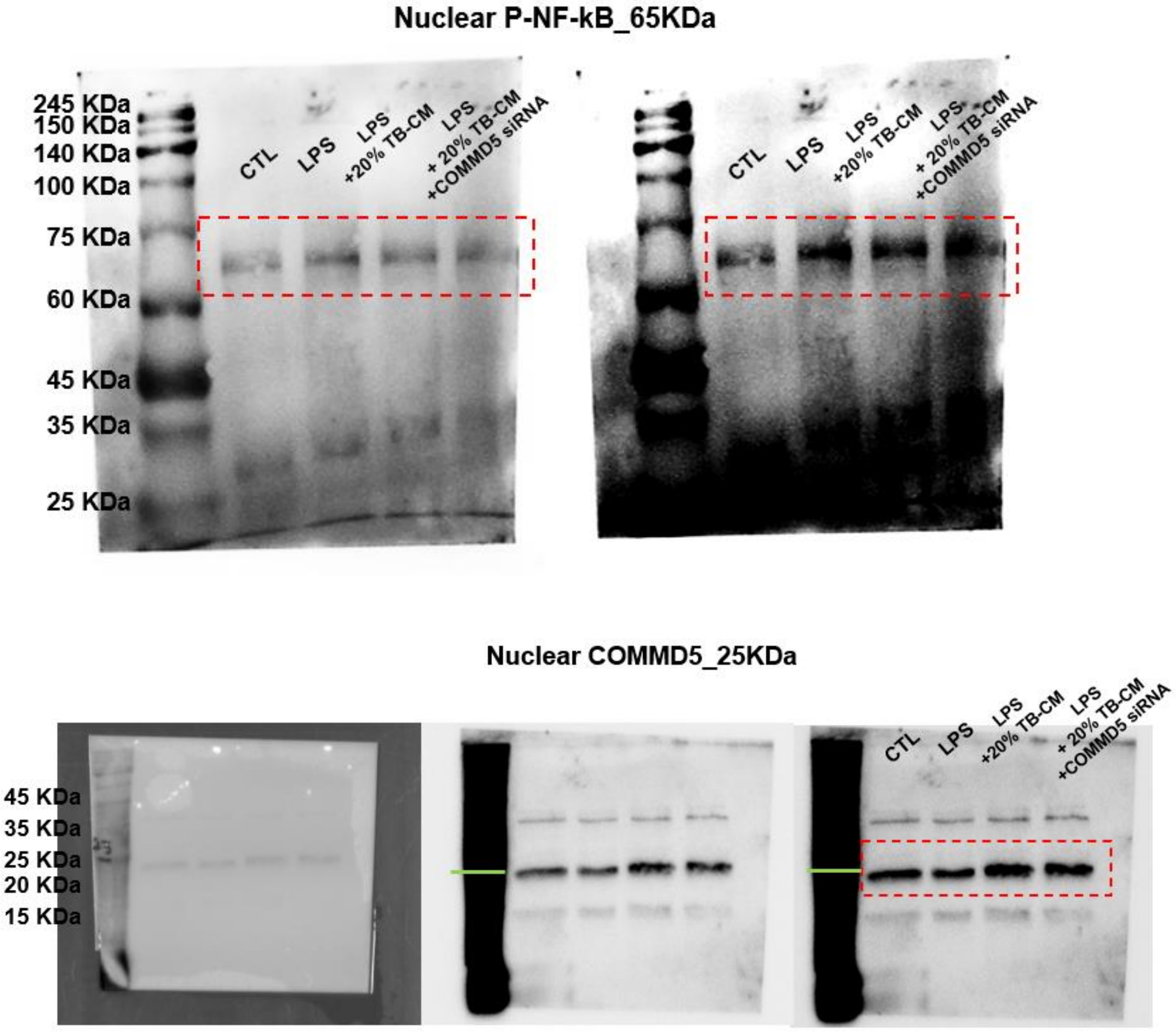
Nuclear P-NF-kB_65KDa
245 KDa
150 KDa
140 KDa
100 KDa
75 KDa
60 KDa
45 KDa
35 KDa
25 KDa
CTL
LPS
LPS +20% TB-CM
LPS + 20% TB-CM +COMMD5 siRNA
Nuclear COMMD5_25KDa
45 KDa
35 KDa
25 KDa
20 KDa
15 KDa

Nuclear β-actin_42KDa

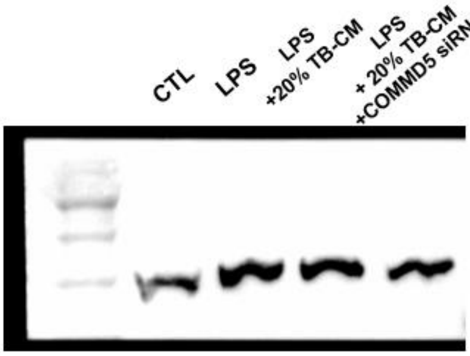

Fig.S2c

ALIX_95 KDa

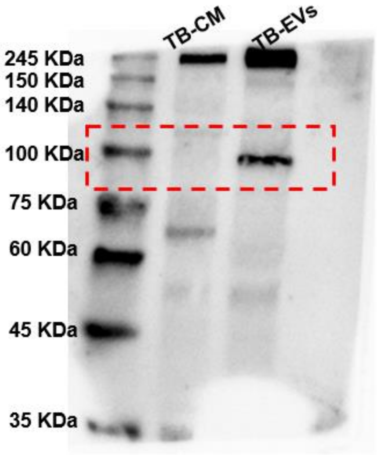

CD63_30-60 KDa

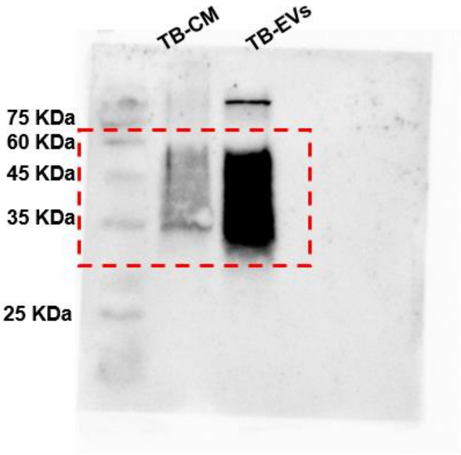

CD81_25 KDa

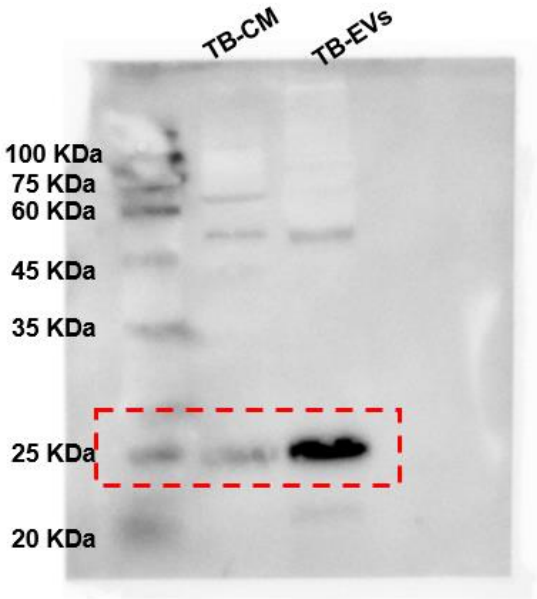

β-actin_42 KDa

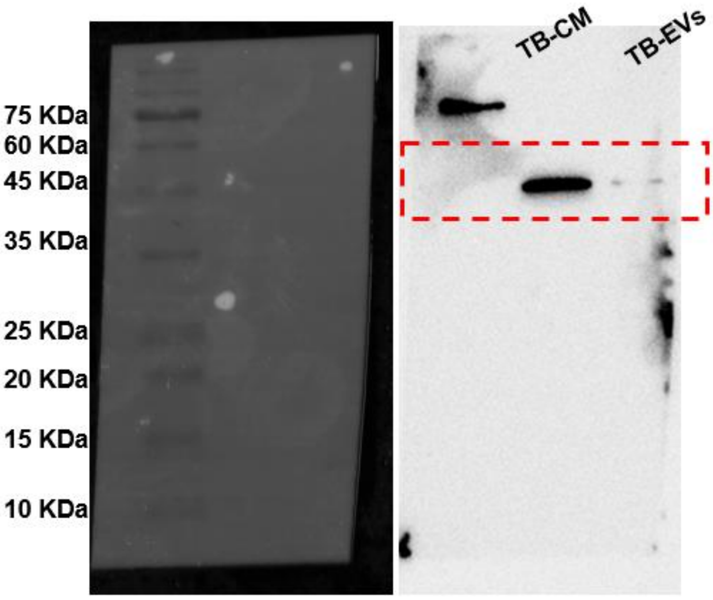

Raw data images of Western blots are shown in Fig. 1e, Fig. 5c, Fig. 5e, Fig. 6a, Fig. 6b, and FigS2c.
